# Predicting Working Memory Training Benefits From Transcranial Direct Current Stimulation Using Resting-State fMRI

**DOI:** 10.3389/fpsyg.2020.570030

**Published:** 2020-10-14

**Authors:** Adelle G. B. Cerreta, Ryan E. B. Mruczek, Marian E. Berryhill

**Affiliations:** ^1^ Program in Cognitive and Brain Sciences, Program in Integrative Neuroscience, Department of Psychology, University of Nevada, Reno, NV, United States; ^2^ Department of Psychology, College of the Holy Cross, Worcester, MA, United States

**Keywords:** working memory, transcranial direct current stimulation, resting-state, fMRI, cognitive training

## Abstract

The effects of transcranial direct current stimulation (tDCS) on working memory (WM) performance are promising but variable and contested. In particular, designs involving one session of tDCS are prone to variable outcomes with notable effects of individual differences. Some participants benefit, whereas others are impaired by the same tDCS protocol. In contrast, protocols including multiple sessions of tDCS more consistently report WM improvement across participants. The objective of the current project was to test whether differences in resting-state connectivity between stimulation site and two WM-relevant networks [default mode network (DMN) and central executive network (CEN)] could account for initial and longitudinal responses to tDCS. Healthy young adults completed 5 days of visual WM training during sham or anodal right frontal tDCS. The behavioral data showed that only the active tDCS group significantly improved over the visual WM training period. There were no significant correlations between initial response to tDCS and resting-state activity. DMN activity in the anterior cingulate cortex significantly correlated with WM training slope. These data underscore the importance of sampling in studies applying tDCS; homogeneity (e.g., of gender, special population, and WM capacity) may produce more consistent data in a single experiment with limited power, whereas heterogeneity is important in determining the mechanism(s) and potential for tDCS-linked protocols. This issue is a limitation in tDCS findings that continues to hamper its optimization and translational value.

## Introduction

Neurostimulation offers hope as a way to improve capacity-limited working memory (WM; Cowan, 2001) and its age-related decline (Reuter-Lorenz and Park, 2010). To date, cognitive training regimens produce modest results with little evidence of transfer to untrained tasks (for reviews see: Morrison and Chein, 2011; Shipstead et al., 2012; Melby-Lervåg et al., 2016; Simons et al., 2016; Katz et al., 2018; Redick, 2019; Sala and Gobet, 2019; Teixeira-Santos et al., 2019; but see: Au et al., 2016a).

When WM benefits are found, the underlying mechanism appears to be enhanced frontoparietal connectivity (McNab and Klingberg, 2008; Constantinidis and Klingberg, 2016; Chen et al., 2019). These networks can be targeted using noninvasive transcranial direct current stimulation (tDCS). Some evidence shows that one session of tDCS can improve WM performance in healthy (Sandrini et al., 2012; Brunoni and Vanderhasselt, 2014; Trumbo et al., 2016; Brunye et al., 2017), and patient populations (Boggio et al., 2006; Jo et al., 2009; Saunders et al., 2015; Wu et al., 2016), reviewed in Berryhill and Martin (2018). In contrast to the variability in single-session protocols, tDCS-WM training protocols show more consistent WM benefits (Martin et al., 2013; Richmond et al., 2014; Jones et al., 2015b, 2017; Au et al., 2016b; Stephens and Berryhill, 2016; Katz et al., 2017; Ke et al., 2019; but see: Möller et al., 2017). However, the jury remains split as several reviews, and meta-analyses provide differing conclusions, suggesting that there are few cognitive benefits associated with tDCS (Horvath et al., 2015), that meta-analyses minimizing tDCS-effects are flawed (Antal et al., 2015), or that there are benefits under certain conditions, such as longitudinal designs (Brunoni and Vanderhasselt, 2014; Elmasry et al., 2015; Hill et al., 2016; Jantz et al., 2016; Mancuso et al., 2016).

We reiterate our stance that one underlying challenge is that individual differences play an important, but poorly understood, role in determining how a participant responds to tDCS. Individual participants show different behavioral effects in response to the same tDCS protocol (Benwell et al., 2015; Brunye et al., 2015; London and Slagter, 2015; Filmer et al., 2019). We previously reported that tDCS effects interacted with individual differences based on participants’ WM capacity (Jones and Berryhill, 2012; Gözenman and Berryhill, 2016; Arciniega et al., 2018), level of educational attainment (Berryhill and Jones, 2012), and even level of motivation (Jones et al., 2015a). Others find that the temporal spacing of sessions (Au et al., 2016b; Katz et al., 2017), brain morphology (Kim et al., 2014), and even genetics (Wiegand et al., 2016) modulate tDCS effects. A few studies examining individual differences in longitudinal designs find evidence of different responses even after multiple sessions (Stephens et al., 2017; Talsma et al., 2017; Ke et al., 2019). Individual differences appear to be more consequential in single-session compared to multiple-session tDCS studies. One persistent difficulty is to sufficiently understand the underlying mechanism of tDCS-linked WM benefits to leverage these differences into tailored, efficacious protocols.

Here, we test the hypothesis that initial resting-state connectivity will predict the initial response as well as the longitudinal response to tDCS in a visual WM task. This hypothesis builds on prior findings showing that tDCS alters synaptic strength in task-relevant networks (Filmer et al., 2014) for a period of time after stimulation (Callan et al., 2016; Jones et al., 2017; Möller et al., 2017; Antonenko et al., 2018; Fiori et al., 2018; Nissim et al., 2019). Furthermore, tDCS targeting the right prefrontal cortex (PFC) revealed strengthened functional connectivity in frontoparietal networks (Peña-Gómez et al., 2012) within the central executive network (CEN; Collette and Van der Linden, 2002; Owen et al., 2005; Seeley et al., 2007; Bressler and Menon, 2010; Peña-Gómez et al., 2012) and other cortical-subcortical networks (Polania et al., 2011). The CEN is a task-positive network with stronger connectivity between dorsolateral PFC (DLPFC) and posterior parietal cortex associated with superior WM performance (Duncan and Owen, 2000; Curtis and D’Esposito, 2003; Bunge and Wright, 2007; Palva et al., 2010). In addition, connectivity in the task-negative default mode network (DMN) is important for WM performance (Hampson et al., 2010; Sambataro et al., 2010; Vatansever et al., 2017) and can predict individual differences in WM performance (Sala-Llonch et al., 2012). tDCS can also modulate DMN connectivity (Keeser et al., 2011; Abellaneda-Perez et al., 2019; Chase et al., 2019; Donaldson et al., 2019; Mezger et al., 2019). Finally, to complete the circle on these two networks, the relationship between the CEN and DMN is related to WM performance (Hampson et al., 2010).

Collectively, these findings raise the possibility that an individual’s pattern of connectivity may predict their response to tDCS. We collected baseline resting-state fMRI (rsfMRI) and conducted a tDCS-WM training study in the same individuals. We predicted that we would replicate the observation that stronger resting-state connectivity in the DMN or CEN would predict higher WM capacity before tDCS. The next prediction related to initial response to tDCS. Our logic was that the subtle effect of one tDCS session would be enough to modulate well-functioning networks in those with higher WM capacity and stronger connectivity. Whereas in low WM capacity participants, we anticipated insufficient connectivity that would require multiple tDCS sessions to improve WM performance. The prediction associated with the more consistent response associated with longitudinal response was that there might be distinct pattern of connectivity that collectively predicted performance gains over multiple tDCS sessions.

## Materials and Methods

### Participants

Forty-six University of Nevada, Reno students participated in exchange for their choice of $15/h or course credit (active tDCS: *N* = 28, ages 18–36, *M* = 22.7, 12 females; sham: *N* = 18 ages 18–32, *M* = 21.4, eight females). Participants had no history of neurological conditions and were not taking any sedative-hypnotic medications that might alter neural excitability. All protocols were approved by the Internal Review Board of the University of Nevada, and informed consent was collected prior to participation. The data from three participants in the active tDCS group were excluded: one participant due to below chance (<50%) performance across *all* sessions and two others for failing to complete all five sessions.

### Resting-State MRI Acquisition and Preprocessing

First, participants completed one rsfMRI session prior to WM testing or any tDCS. Participants kept eyes closed during 2–3 rsfMRI runs (~5.3 min each); 14 active group members completed two runs, and the remaining participants completed three runs. Images were acquired on a 3 T Philips (Andover, MA, United States) MRI scanner with an eight-channel SENSE parallel head coil. A set of 155 T2^*^-weighted volumes were obtained [repetition time (*TR*) = 2,000 ms, echo time (*TE*) = 30 ms, 32 slices per volumes, slice thickness = 3 mm, field of view (FOV) = 240 mm^2^, matrix size 128 × 128, in-plane resolution = 1.875 mm]. Functional data were aligned to a high-resolution 3D structural dataset using an echo-planar 3D T1-weighted image.

Preprocessing was completed in analysis of functional neuroimages (AFNI; Cox, 1996; http://afni.nimh.nih.gov/afni/), SUMA (Saad et al., 2004; http://afni.nimh.nih.gov/afni/suma/), and FreeSurfer (Dale et al., 1999; Fischl et al., 1999; http://surfer.nmr.mgh.harvard.edu/) using the standardized script *afni_proc.py.*^1^ The first two TRs were removed, then the data were despiked, and slice-time and motion were corrected, as well as spatially normalized to an Montreal Neurological Institute (MNI) template. Bandpass filtering was employed to temporally filter and retain frequencies between 0.01 and 0.1 Hz. Censoring was based on motion parameters and signal outliers within the BOLD data (Power et al., 2014, 2015). Six motion parameter estimates, ventricular and white matter signals, and baseline, linear, quadratic, and cubic trends were removed by linear regression (Fox et al., 2009).

### Regions of Interest and Producing Seeds

Seeds were restricted to right hemispheric areas, as we used spatial WM tasks. Seed regions of interest (ROIs) were manually generated in AFNI. The seeds were 12 mm spheres to allow for broader connectivity maps and to be consistent with existing guidelines (Fox et al., 2005). The following seed locations were selected: (1) right DLPFC, a node in the CEN for correlations related to executive functioning during resting-state (MNI coordinates: 44, 36, and 20; Seeley et al., 2007); and (2) right posterior cingulate cortex (PCC) within the DMN to acquire canonical resting-state activity maps (MNI coordinates: 5, −49, and 40; Fox et al., 2005).

### Resting-State Analysis

To determine connectivity strength between these seed regions and the rest of the brain, AFNI’s *3dUndump* created the ROI by generating a 3D dataset from the specified coordinates. *3dmaskave* was used to generate the time course of activity in the given seed region. To determine correlation values, *3dfim*+ was used to correlate time course correlations within either the DMN seed (right PCC) or CEN seed (right DLPFC) and behavioral performance. *3dfim*+ was also used to correlate the DMN and CEN time courses with time courses throughout the whole brain, generating resting-state connectivity maps of Pearson’s *r* values. Pearson’s *r* values were converted to *z*-scores using Fisher’s *r*-to-*z* transformation and the expression “*log[(1* + *a)/(1* − *a)]/2*.” In SPSS (version 24, IBM, Armonk, NY, United States), *z*-scores, and performance scores for each individual were compared across all participants, as well as by high and low WM capacity groups (see below), to identify significant differences in hemodynamic activity and performance.

### Transcranial Direct Current Stimulation

**Figure 1:**
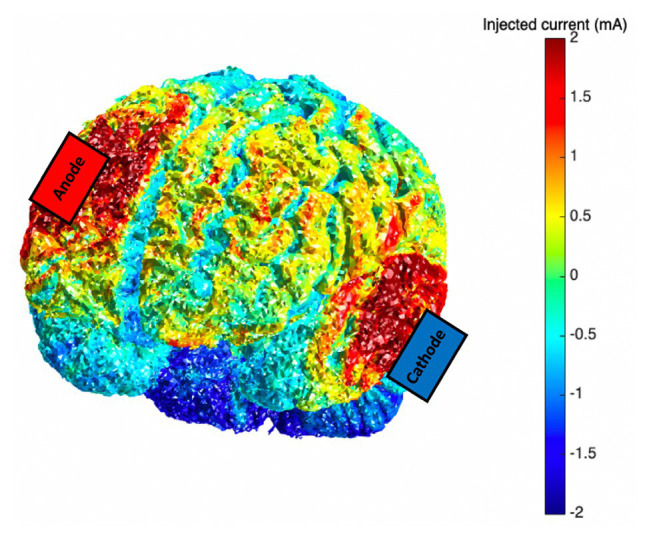
Current modeling for the transcranial direct current stimulation (tDCS) montage (anode: F4; cathode: contralateral cheek); 2.0 mA tDCS was applied *via* scalp-based electrodes.

Anodal tDCS stimulation was applied *via* two saline-dampened scalp electrodes (5 × 7 cm^2^) and delivered at 2 mA for 20 min by a continuous current stimulator (neuroConn DC Stimulator, GmbH, Germany). The anode was centered over the right (F4) DLPFC. The cathode was placed on the contralateral cheek (Berryhill et al., 2010; Jones et al., 2015b). Sham stimulation also involved placement of electrodes in these areas, but unbeknownst to the participant, only brief (20 s ramp up/down) stimulation was applied. Current modeling was also investigated using the Realistic vOlumetric Approach to Simulate Transcranial Electric Stimulation (ROAST, Huang et al., 2019; see Figure 1).

### Behavioral Paradigms

On Day 1 of the five-session WM training period, participants completed the Automated Operation Span (OSPAN) to provide an independent baseline measure of WM performance (Unsworth et al., 2005), prior to any tDCS. Participants remembered the order of a string of sequentially presented letters interleaved with arithmetic problems that also required WM.

Participants received tDCS during WM training tasks performance. Participants completed three tasks: a change detection task (set sizes 6 and 8) and a two-back visuospatial task (described below). In the change detection tasks, participants viewed six- or eight-colored squares (250 ms, 3 × 3°, white, black, red, pink, orange, yellow, green, teal, blue, aqua, and purple; Jones and Berryhill, 2012). After a delay (1,000 ms) a probe image (2,200 ms) appeared that was unchanged from encoding (50%) or included one color change (50%). Participants responded *via* key press (“o” match and “n” mismatch). There were 100 trials per set size, and the task lasted ~10 min. The change detection task was used because although attention is needed for the task, WM is crucial for accessing the representation stored during encoding for comparison. In the spatial *n*-back task participants monitored the location of a green circle (500 ms) across nine possible locations (Stephens and Berryhill, 2016). During each presentation, participants reported *via* key press (“j” match and “f” mismatch) whether the current location matched the location occupied two presentations previously. Participants completed 450 trials (150 match and 300 no match) in ~10 min.

The primary measure for each task was percent accuracy. Percent accuracy on the two-back task was calculated for each day individually, with analyses geared toward revealing any significant differences in percent accuracy dependent on session number. The same analyses were completed for the change detection task with set sizes 6 and 8, individually. Subsequently, performance slope over the five sessions was also considered to further investigate improvement.

## Results

### The *Active*-tDCS Group Improved on the Two-Back Task With Training

**Figure 2:**
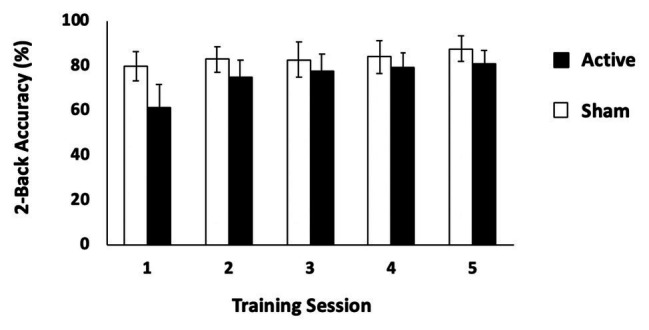
Behavioral results: two-back task. Performance (% correct) on the two-back task for the active and sham tDCS stimulation conditions. Error bars represent 95% CIs.

The first question to address was to determine whether active tDCS significantly changed WM performance compared to sham stimulation. We first conducted separate repeated measures ANOVAs with the factors of group (active tDCS and sham) and session (Days 1–5) for each WM training task. This revealed that neither group significantly improved on *either* of the change detection tasks [set sizes 6 and 8; set size 6: *F*(4,168) = 2.04, *p* = 0.09, *η*_p_^2^ = 0.05; set size 8: *F*(4,164) = 0.96, *p* = 0.43, *η*_p_^2^ = 0.02]. Performance on the change detection task was poor and varied little across sessions (set size 6: active: Day 1: mean, SD: 65.68, 7.95%; Day 5: 68.20, 7.37%; sham: 66.00, 7.39%, Day 5: 69.18, 7.04%; set size 8: active: Day 1: 62.16, 6.44%; Day 5: 62.84, 7.15%; sham: Day 1: 61.13, 7.04%; Day 5: 63.06, 4.52%). However, performance on the two-back task revealed a significant group × session interaction indicating that *only* the active group significantly improved across sessions [*F*(4.88,75.66) = 7.15, *p* < 0.001, *η*_p_^2^ = 0.16; Greenhouse-Geisser correction applied for violation of sphericity; see Figure 2, but this effect did not survive when performance on Day 1 was entered as a covariate *F*(1,40) = 0.54, *p* = 0.47]. A complementary analysis of slope across training sessions provided confirmation that the active tDCS group improved more steeply than did the sham group [sham: mean slope (*M*) = 0.06, 95%*CI* = (−0.47, 0.60); active: *M* = 3.36, *CI* = (1.75, 4.97); independent-samples *t*-test, *t*(31.70) = 2.96, *p* = 0.006, *η*^2^ = 0.23; Levene’s test: *p* = 0.004]. This effect did survive an ANCOVA with Day 1 performance as a covariate [*F*(1,40) = 7.00, *p* = 0.012]. In sum, only the *active* tDCS group showed significant improvement in WM performance over the 5 days of training.

Reaction times on the two-back task were investigated, but no significant difference in reaction time performance was found between active and sham groups [Greenhouse-Geisser corrected, *F*(2.146,87.968) = 0.10, *p* = 0.92].

### Active tDCS Group: Predicting Training Response to tDCS

#### Resting-State fMRI Does Not Predict Initial Response to tDCS (Day 1 Response)

With regard to the rsfMRI data, we were interested in the relationship between *pre-existing* rsfMRI and an *individual’s* WM response to initial and longitudinal tDCS. We planned on probing rsfMRI within the DMN and CEN to test whether connectivity in these networks predicted the initial and/or later responsivity to tDCS. The logic was that this might be useful in predicting tDCS-linked WM performance. There were *no* significant correlations reflecting initial response to tDCS on Day 1 of WM training (two-back task) and either rsfMRI seed. In other words, we found no support for our hypothesis that resting-state differences in connectivity would predict initial response to tDCS.

#### Resting-State fMRI Does Predict Longitudinal tDCS Response (Training Slope)

**Figure 3:**
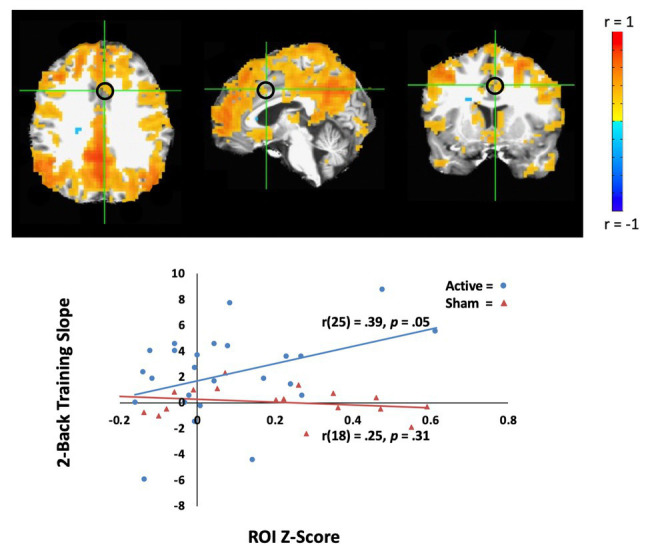
Resting-state fMRI (rsfMRI) activity correlates with behavior learning across the training in the active tDCS group. **Top**: The average of default mode network (DMN) activity (whole brain connectivity generated from the PCC seed and circled in black) in the active tDCS group participants. The left hemisphere is shown on the left. **Bottom**: Training slope on the two-back task significantly correlated with DMN activity in the anterior cingulate cortex [Montreal Neurological Institute (MNI) seed: (4, 12, and 36), black circle]. The sham group participants are shown as orange triangles, and the active group participants are shown as blue circles.

In the active tDCS group, we investigated whether rsfMRI predicted WM performance across tDCS-linked WM training. To address this question, we sought correlations between training slope on the two-back task and rsfMRI correlations with two networks, the DMN, and CEN. For the CEN seed, there was no significant correlation that predicted WM performance slope. However, the connectivity between the DMN seed (PCC) and the anterior cingulate cortex positively correlated with WM performance slope for the active group [Pearson’s *r*(25) = 0.39, *p* = 0.05, MNI coordinates: (4, 12, and 36); see Figure 3], but not for the sham group [*r*(18) = 0.25, *p* = 0.31]. DMN connectivity before training predicted active group gains in the 2-back task.

## Discussion

In this experiment, WM training, tDCS, and rsfMRI measures were combined to clarify the basis of individual differences in tDCS-linked WM response. We tested whether pre-existing connectivity predicted initial or longitudinal responses to tDCS-linked cognitive performance. Participants completed change detection and *n*-back WM tasks across five training sessions with or without active right frontal tDCS. The data showed that only the active tDCS group significantly improved. Improvement was isolated to performance on the two-back task as performance on the change detection task was consistent across sessions.

Notably, there were no patterns of resting-state connectivity that corresponded with Day 1 responses to tDCS. This held across both seed locations, one in the DMN and one in the CEN. These data provide no support for our intuition that connectivity could characterize the effects of individual differences after a single session of tDCS. As noted above, we have previously seen data that conform to a phenomenon of the “rich get richer” such that performance improvements occur in the subset of participants with high WM capacity (Jones and Berryhill, 2012) or high education level (Berryhill and Jones, 2012). If existing connectivity patterns do not explain initial responses to tDCS, we speculate that neural excitability during tDCS explains immediate effects. In short, it has been found that those who show greater increase in BOLD during anodal parietal tDCS demonstrate superior performance in a visual navigation task (Falcone et al., 2018). Alternatively, the effect of connectivity may be quite small, and only detected with more participants, or higher resolution scanning.

In contrast, two-back WM training slope was predicted by the coupling strength between the DMN seed and the anterior cingulate cortex. After WM training, resting-state scans have shown that the anterior cingulate cortex exhibits increased functional connectivity with frontoparietal networks, with personality indices correlating with changes in anterior cingulate cortex activity (Gray and Braver, 2002; Jolles et al., 2013). Lesions to this area impair WM ability in identifying errors and executing a corrective action (Rushworth et al., 2003). Our interpretation is that the tDCS builds on existing connectivity to further improve WM performance. It predicts that individuals with well-developed connectivity between these areas may benefit more from longitudinal tDCS than those with less. In other words, the current analyses may have identified the tips of the icebergs, and further work is needed to clarify those who will benefit from tDCS.

Assuredly, with more rsfMRI scans and more participants, we may be better able to identify more subtle relationships. It is now quite clear that tDCS provides a modest neuromodulatory effect that serves as a tipping factor to alter neural firing. A major challenge in going forward in developing effective tDCS protocols for cognition or clinical populations is that in addition to individual differences such as connectivity, there are many factors and stimulation parameters that predict an individual’s response to tDCS. These variables include neural excitability, amount of sleep, engagement with the task, genotype (see Katz et al., 2017 for review).

## Limitations

A major limitation is that our participants exhibited low heterogeneity in their WM performance than in our participant samples in previous studies. Because we were conducting resting-state scans paired with a longitudinal tDCS design, we worried about attrition, and consequently, we recruited from department affiliates (e.g., graduate students and research assistants). This meant that we were drawing from a homogeneous pool and a group that was strongly motivated to provide quality data. Thus, solving one problem raised a second, unanticipated challenge: less range in our participants’ performance, especially in the sham group. Additional differences in connectivity might be more nuanced or clearer in a more representative population. In addition, connectivity patterns in older adults change over time (Chan et al., 2014), and what may be useful in predicting tDCS responsiveness in young adults may differ in the elderly. These issues served to weaken our statistics and render null the difference between the active and sham group correlations.

A second limitation that reduced generalizability was that the change detection tasks showed no improvement across sessions, possibly due to a lack of engagement with the task and leading us to eliminate these data from further analyses. We implemented multiple tasks to address generality of any observed effects, and we did not want our participants to be bored with many trials of the same task over multiple days. Finally, we would like to have been able to collect more resting-state scans per person to have cleaner, more powerful data. A major advantage to using rsfMRI over task-related fMRI is the reduced time and number of scans necessary compared to when typically using a task. More subjects can be used, and sensitive subjects do not have to be in the scanner for as long. An additional possibility is that despite existing findings associating rsfMRI with task performance, it may not be as sensitive to subtle changes elicited by tDCS. In other words, task-related fMRI may be superior in these situations. Future work with more powerful rsfMRI protocols is essential in building the optimized, tailored tDCS protocol to maximize performance in an efficient manner.

## Open Questions

TDCS may yet prove to be a generally useful translational approach. Converging evidence supports the importance of neural synchronization to change neural networks during WM task performance. A major challenge is to determine *a priori* who respond to stimulation. Individual differences are an acknowledged aspect of the noninvasive stimulation literature and help to explain inconsistent results. Converging evidence from various techniques, including further testing of tDCS parameters, morphological and functional differences in the brain, and genetic differences are needed.

## Data Availability Statement

The raw data supporting the conclusions of this article will be made available by the authors, without undue reservation.

## Ethics Statement

The studies involving human participants were reviewed and approved by University of Nevada, Reno IRB. The patients/participants provided their written informed consent to participate in this study.

## Author Contributions

AC and MB designed the study. AC collected the data. AC and RM completed analyses. All authors wrote the manuscript. All authors contributed to the article and approved the submitted version.

### Conflict of Interest

The authors declare that the research was conducted in the absence of any commercial or financial relationships that could be construed as a potential conflict of interest.

## Footnotes

^1^http://afni.nimh.nih.gov/pub/dist/doc/program_help/afni_proc.py.html
